# Evolution of knowledge on meniscal biomechanics: a 40 year perspective

**DOI:** 10.1186/s12891-021-04492-2

**Published:** 2021-07-15

**Authors:** Amin Mohamadi, Kaveh Momenzadeh, Aidin Masoudi, Kempland C. Walley, Kenny Ierardi, Arun Ramappa, Joseph P. DeAngelis, Ara Nazarian

**Affiliations:** 1 Musculoskeletal Translational Innovation Initiative, Beth Israel Deaconess Medical Center, Harvard Medical School, 330 Brookline Avenue, RN123, Boston, MA 02215 USA; 2 Carl J. Shapiro Department of Orthopaedic Surgery, Beth Israel Deaconess Medical Center, Harvard Medical School, Boston, MA USA; 3 Department of Orthopaedic Surgery, Yerevan State Medical University, Yerevan, Armenia

**Keywords:** Meniscus, Biomechanics, Osteoarthritis, Systematic review, Mechanical stress

## Abstract

**Background:**

Knowledge regarding the biomechanics of the meniscus has grown exponentially throughout the last four decades. Numerous studies have helped develop this knowledge, but these studies have varied widely in their approach to analyzing the meniscus. As one of the subcategories of mechanical phenomena Medical Subject Headings (MeSH) terms, mechanical stress was introduced in 1973. This study aims to provide an up-to-date chronological overview and highlights the evolutionary comprehension and understanding of meniscus biomechanics over the past forty years.

**Methods:**

A literature review was conducted in April 2021 through PubMed. As a result, fifty-seven papers were chosen for this narrative review and divided into categories; Cadaveric, Finite element (FE) modeling, and Kinematic studies.

**Results:**

Investigations in the 1970s and 1980s focused primarily on cadaveric biomechanics. These studies have generated the fundamental knowledge basis for the emergence of FE model studies in the 1990s. As FE model studies started to show comparable results to the gold standard cadaveric models in the 2000s, the need for understanding changes in tissue stress during various movements triggered the start of cadaveric and FE model studies on kinematics.

**Conclusion:**

This study focuses on a chronological examination of studies on meniscus biomechanics in order to introduce concepts, theories, methods, and developments achieved over the past 40 years and also to identify the likely direction for future research. The biomechanics of intact meniscus and various types of meniscal tears has been broadly studied. Nevertheless, the biomechanics of meniscal tears, meniscectomy, or repairs in the knee with other concurrent problems such as torn cruciate ligaments or genu-valgum or genu-varum have not been extensively studied.

## Background

The knee joint menisci are crescent-shaped fibrocartilaginous soft tissue, which provides significant biomechanical functionalities within the knee joint. These semilunar-shaped structures enable the knee joint to move in all six degrees of freedom and are essential for load transmission and distribution, shock absorption, and knee joint lubrication and stabilization [1–4]. “Mechanical stress” was first defined as one of the subcategories of “Mechanical Phenomena” MeSH terms in 1973. Mechanical stress quantitatively describes a purely physical status within a material in response to an external force over a given area quantified by units of force per unit area. Most commonly, given stress is associated with deformation or strain (a relative change compared to the initial status before the stress was experienced).

It was not that long ago when menisci were thought to be functionless embryonic residue [5]. Nowadays, injuries to the menisci are recognized as a cause of significant musculoskeletal morbidity and an important cause of knee osteoarthritis [6]. Vast improvements in the overall understanding of meniscus biomechanics have been made over the past forty years. Scientific exploration of meniscal biomechanics began in 1971, focusing on using cadaveric models [7]. These cadaveric models built the foundation for the emergence of FE (Finite Element) model studies in the 1990s [8–10]. By the 2000s, FE models showed results comparable to the gold standard cadaveric models [10–12]. This achievement brought about the opportunity to combine these two methods, which provided a more in-depth understanding of the meniscus. Kinematic studies have now emerged to improve our knowledge of meniscal tissue behavior, applied stress, and physical movement.

The purpose of this study was to provide a chronologic overview of the evolutionary comprehension and understanding of biomechanical properties of the menisci over the past forty years. The review contains a concise and detailed description of cadaveric, FE, and kinematic studies concerning knee biomechanics. Ultimately, an improved understanding of meniscus biomechanics could lead to better study designs in the future to address the existing gaps of knowledge in this field.

## Main text

### Study selection

A literature review was conducted through PubMed (National Library of Medicine and National Institute of Health, USA) in April 2021, focusing on evaluating stress for meniscal tissue. Studies published in the English language, conducted on human menisci, focused on mechanical stress, and published in peer-reviewed journals were eligible for review. Studies evaluating the animal subjects, case reports, review articles, influence of biologic factors, repair techniques, discoid meniscus, or pathologies other than meniscal tear were excluded from this review.

Fifty-seven papers were included to provide a chronologic overview of the evolutionary comprehension and understanding of meniscus biomechanics over the past forty years. To most efficiently explore this topic, this review focused on the three following characterizations of biomechanical investigation types: (1) Cadaveric, (2) FE Analysis, and (3) Kinematic investigations.

### Cadaveric studies

**Fig. 1:**
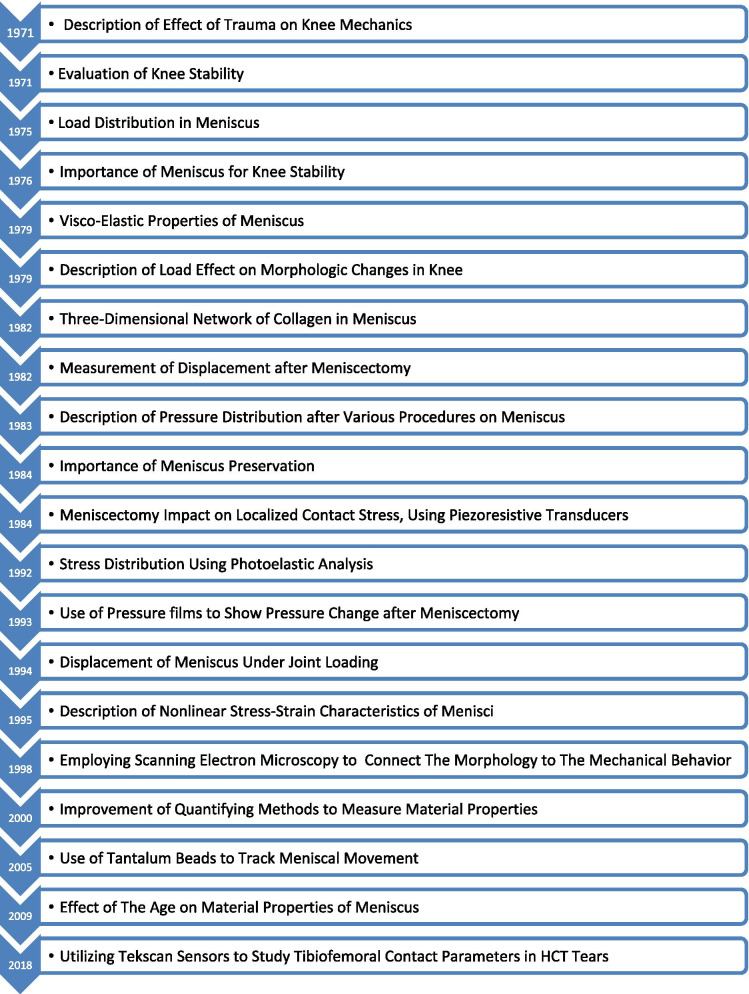
Significant events in cadaveric studies of the meniscus

In English literature, it was not until 1971 that the meniscus was reported as an essential structure in the knee concerning overall joint biomechanics [7]. Cadaveric specimens naturally allowed for empirical investigations to take place, through which the fundamentals of meniscal biomechanics were incrementally understood over a span of nearly 50 years (Fig. 1). Due to cadaveric studies’ extensiveness, in this review, studies are presented in three subcategories, A) Displacement and Joint Stability, B) Collagen Fiber, Viscoelasticity, and Shock Absorption, and C) Response to Load, Compression, and Tension.

#### Displacement and joint stability

**Fig. 2:**
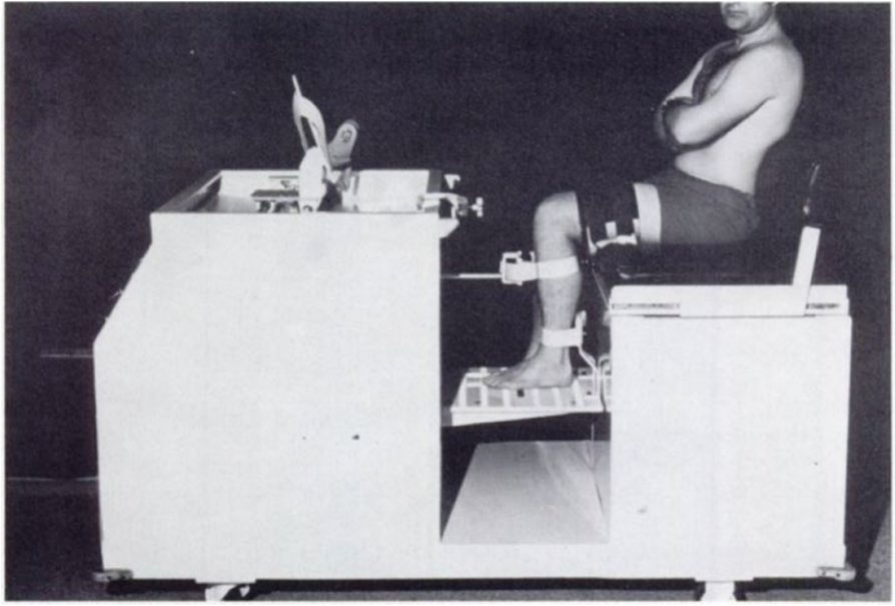
Clinical stress machine for evaluating the role of the intact meniscus and its influence on tibiofemoral stability. From “Medial and anterior instability of the knee. An anatomical and clinical study using stress machines” by Kennedy JC, Fowler PJ, 1971, The Journal of bone and joint surgery American volume. 1971;53(7):1257–70 [13]. With permission of Wolters Kluwer. Promotional and commercial use of the material in print, digital or mobile device format is prohibited without the permission from the publisher Wolters Kluwer. Please contact healthpermissions@wolterskluwer.com for further information

In 1971, Frankel et al. showed that trauma caused internal derangement, which led to the displacement of the instant center of rotation, mechanical articular surface wear, and finally, degenerative joint disease [7]. Also, a clinical stress machine was developed in that year to evaluate knee stability before and after meniscectomy (Fig. 2) [13]. Five years later, the importance of the meniscus as an intrinsic factor for stabilizing the knee was shown through a similar biomechanical design modified by the use of imposed cyclic loading, determining anterior–posterior and rotary laxity [14].

Bylski-Austrow et al. investigated kinematic information under loading conditions and quantified the meniscus’ displacement under joint compression coupled with internal–external torques and anterior–posterior static forces at fixed angles of flexion. The lateral meniscus’ displacements were larger than those of the medial meniscus with internal rotation and posterior tibial translation at full extension. Additionally, they demonstrated that the lateral meniscus displaced more at 5° and 30° of flexion under compression than at 0**°** of internal rotation [15]. Ikeuchi et al. introduced a new method to measure meniscal displacement more precisely in 1998, with a lower limit for detection of 10 microns using a specialized needle tip fixed to a target point in the meniscus determining axial displacement via a laser sensor [16].

**Fig. 3:**
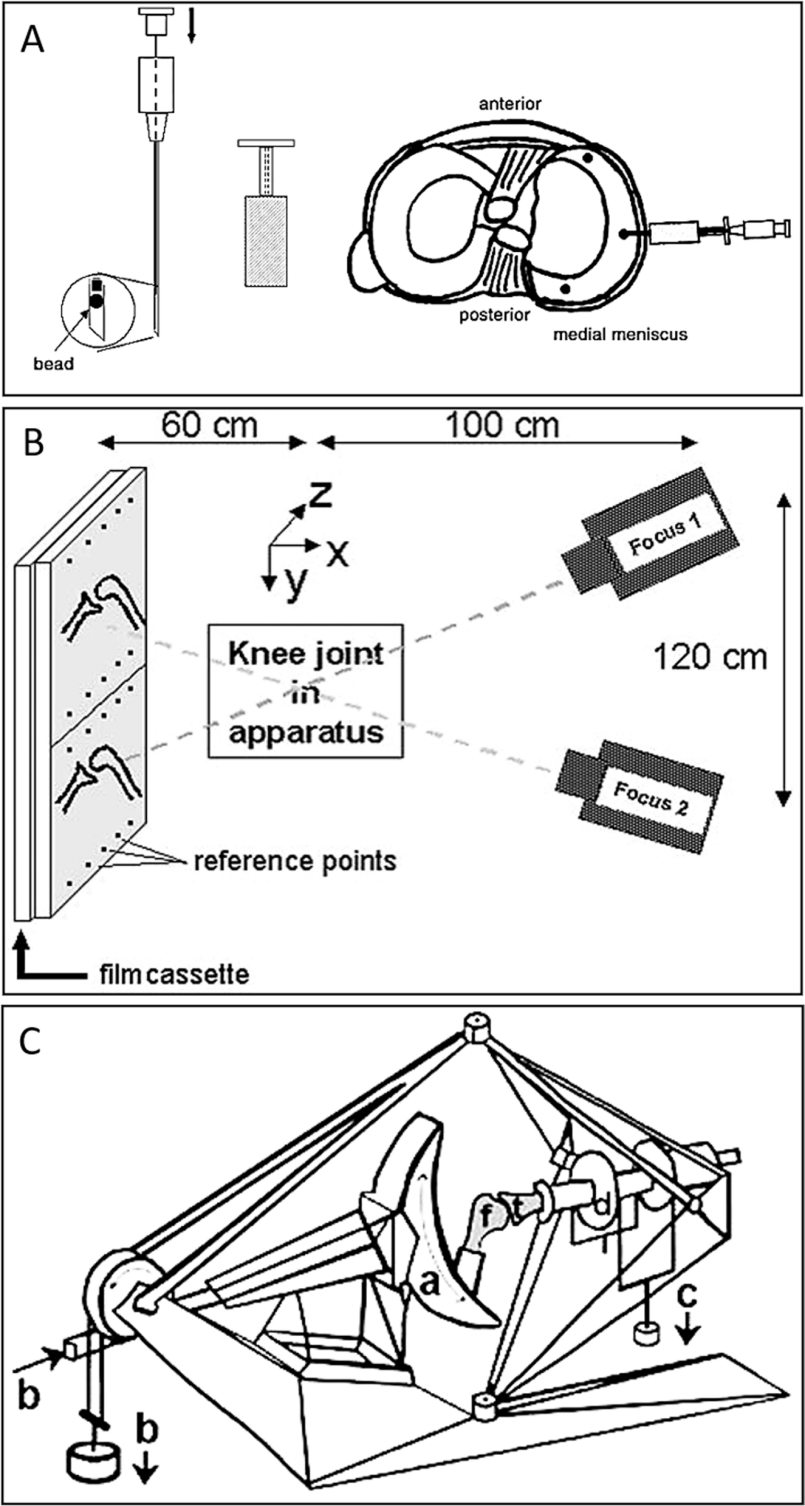
Medial meniscus displacement during passive motion. **A** Schematic view of the insertion device and procedure of insertion of the beads into the meniscus. Beads were placed in the insertion needle, and the needle was again placed into a device that secured an insertion depth of 10 mm in the meniscal stroma through the knee joint capsule. Then, three beads of 0.8 mm diameter were inserted under arthroscopic control. **B** Knee-joint loading apparatus. On the left side, the femur (f), which is rigidly fixated in the semi-lunar device. Different knee-joint flexion angles are realized rotating the semi-lunar device thereby changing the angle of the femur relative to the tibia, as indicated in (a). The axial load (b) is applied to the femur. On the right side, the tibia (t). On this side of the apparatus, freedom of movements are internal and external rotation, varus–valgus rotation, ML translation and AP translation. Internal and external torques (d) were applied through a pair of sheaves (c). **C** Positioning of the loading apparatus, the Roentgen tubes, and the film cassette. Two separate X-rays were taken with two tubes on the same Roentgen film. Note the reference points on the film cassette and the positioning of the knee joint in the coordinate system: the X-direction represents ML displacements on the tibial plateau and the Z-direction represents the AP displacement on the tibial plateau. From “Displacement of the medial meniscus within the passive motion characteristics of the human knee joint: an RSA study in human cadaver knees.” by Tienen TG et al., 2004, Knee Surg Sports Traumatol Arthrosc. 2005;13(4):287-92 [17]. With permission of Springer

In 2005, a cadaveric model was utilized to evaluate meniscal kinematics during knee flexion compared to corresponding results from in-vivo meniscal movement studies (Fig. 3). This study showed the relative immobility of the posterior horn of the medial meniscus during application of tibial torque, and that the posterior displacement of the pathway on the tibial plateau throughout flexion from 0**°** to 30**°** may be restricted by the attached knee-joint capsule or the femoral condyle [17].

#### Collagen fiber, viscoelasticity, and shock absorption

A comparison of the viscoelastic properties between intact and torn human menisci was conducted via stress relaxation tests with tensile loading by Uezaki et al. in 1979. This study showed similar viscoelastic properties between intact and torn conditions. The authors observed that the strain rate dependence of Young’s modulus increased moderately, providing contrary evidence to the thought that the meniscus primarily acts as a shock absorber in the knee joint [18]. The role of collagen fibers in meniscal tissue and their spatial arrangement in transmitting forces were studied in 1982 by Egner et al. through a comprehensive, histological examination of approximately 4,000 knee joint meniscectomy specimens to establish an understanding of the structure, development, and possible diagnostic criteria. This study established a three-dimensional network of collagen fibers as the mechanically efficient element of the meniscus. This three-dimensional network is characterized by longitudinal fibers observed to assure tension resistance, while transverse and diagonal fiber bundles complement the longitudinal fibers acting as tension rods. This study collectively explained how the meniscus aids in axial compressive resistance [19]. Petersen and Tillman analyzed the meniscus’ collagenous fibril texture to provide a mechanical explanation for meniscal tears’ direction using scanning electron microscopy. They found three distinct layers in the cross-section of the meniscus, of which the circular orientation of the collagen fibrils in the central portion provided a functional explanation for the orientation of meniscal tears [20].

In 2009, Bursac et al. showed that collagen content, proteoglycan content, and tensile properties of menisci from donors younger than 45 years old were not age-dependent [21]. Shock absorption properties of fibrous cartilage in the meniscus were later studied in 2015 [22]. The authors observed that the dynamic modulus of elasticity in hyaline cartilage was approximately ten times higher than that in the meniscus, while the loss angle in fast indentation stayed at the range of hyaline cartilage. This finding showed that hyaline cartilage is more shock absorbing and energy dissipating than the meniscus.

#### Response to load, compression, and tension

Detailed biomechanical information describing the load distribution in the meniscus during axial compressive loading was published in 1975 by Walker et al., who employed a “spatial location” method. This method was used to determine the load-bearing and surface contact areas between the upper tibia and femoral condyle at various flexion angles under conditions with and without load. This group concluded that for applied loads under 150 kg, the medial side’s stress was shared approximately equally by the exposed cartilage and the meniscus. On the lateral side, it appeared that the meniscus carried a majority of the load. Overall, this study concluded that tibiofemoral stability and the knee’s load-bearing areas were enhanced by the menisci [23]. In 1983, Ahmed and Burke revealed that a significant fraction of the joint compressive load was transmitted through the meniscus and that meniscectomy drastically altered the pressure distribution on the tibial surface [1].

The protective role of the meniscus on the articular cartilage of the knee was further investigated through photoelastic methods by Radin et al., who examined the magnitude and distribution of stresses experienced in the medial compartment following meniscectomy, longitudinal meniscal tear, and retention of the meniscal rim. The results from this study reinforced the concept that the meniscus protects the articular cartilage of the knee from localized stress and, therefore, retention of a torn, non-displaced meniscus or the presence of an outer meniscal rim are biomechanically preferable to the absence of the meniscus [24]. In that same year, Bourne et al. compared the strain distribution before and after partial or total medial meniscectomy by simulating an axially loaded single-legged stance. The authors observed changes in the strain on the tibia’s cortical bone, indicating that medial meniscectomy reduced the compressive strains on the lateral aspect of the tibia. In contrast, it increased the compressive strains beyond seventy millimeters distal to the joint-line on the tibia’s medial aspect. However, there was a significant reduction in the compressive strains on the medial aspect within fifty millimeters of the joint-line [25].

**Fig. 4:**
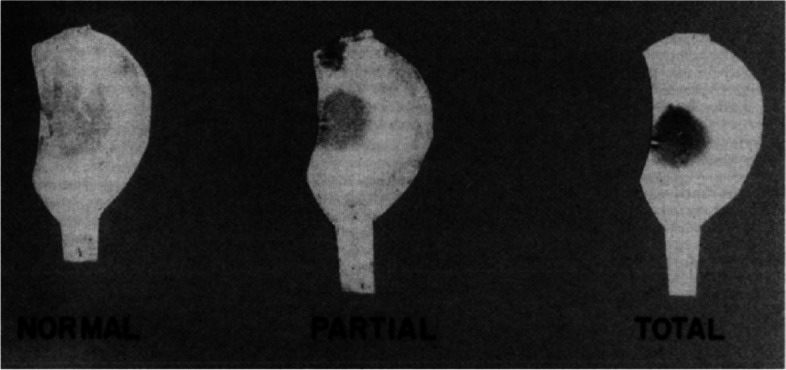
Distribution of pressure on medial tibial plateau total and partial meniscectomy. A total meniscectomy increased peak local contact stresses more than two times and decreased in contact area by 75%, while partial meniscectomy only increased peak local contact stresses and decreased contact areas by 65 and 10%, respectively. From “Meniscal tears: the effect of meniscectomy and of repair on intraarticular contact areas and stress in the human knee. A preliminary report.” by Baratz ME, Fu FH, Mengato R., 1986, Am J Sports Med. 1986;14(4):270–5 [27]. Copyright © 1986 by American Journal of Sports Medicine. Reprinted by Permission of SAGE Publications, Inc

Intuitively, as the amount of cartilaginous tissue protecting the tibial articular surface deteriorates, one would expect that the resulting axial compressive forces would be translated by the femoral condyle to localize on the region of contact; similar to a singular point-contact. This concept was demonstrated by Brown et al., who placed piezoresistive transducers in the cartilage of the femoral condyles and measured the local stress magnitudes of the tibiofemoral joint. They observed that flexion up to 30° did not significantly change the major contact parameters in a normal knee with an intact meniscus. However, with medial and bilateral meniscectomies, the involved contact area reduced, consequently increasing the stress on the cartilage of the femoral condyles. This increased stress was less prominent than what was seen for the static loading of the tibial plateau [26]. Baratz et al. followed this concept in 1986 by comparing the effect of total and partial meniscectomy on peak local contact stresses or simply contact pressure (Fig. 4) [27].

Ihn et al. further studied the effect of centralization of maximum stress concentration points after total meniscectomy on the degenerative process of the knee using a three-dimensional photoelasticity model [28]. In 1993, the same group employed pressure films in a cadaveric model to characterize the role of post-meniscectomy mechanical factors that result in stress concentration. They showed that when all or a part of the meniscus was excised, the stress concentration in the contact area significantly increased. Moreover, they observed that partial meniscectomy decreased the contact area on the joint’s contralateral side, with a more significant decrease after total meniscectomy [29]. Tissakht and Ahmad determined nonlinear stress–strain characteristics of the human meniscus in 1995 using uniaxial elongation tests employing circumferential and radial specimens from various locations, layers, and regions. Importantly, regression analysis from this data demonstrated that only three parameters, (1) the elastic modulus, (2) the maximum strain, and (3) the strain intersect are sufficient in describing the nonlinear stress–strain relationship up to failure [12].

In 2000, Lechner et al. showed that the medial meniscus’ circumferential tensile modulus is affected by the cross-sectional area and the test sample location. This finding provided support for the need to consider these factors when quantifying the material properties of the meniscus [30]. Leslie et al. measured axial, radial, and circumferential compressive forces on meniscal tissue in 2000, who reported that meniscal tissue was significantly stiffer in the axial direction than in the radial and circumferential directions [31]. This finding demonstrates that the meniscus exhibits stronger material properties in the superior-inferior direction (axial) than other planes, in which it is not loaded as often or as intensely. As the extent of meniscectomy that could lead to clinically significant outcomes was uncertain, studies exploring segmental meniscectomy were conducted. In a 2006 cadaveric study by Lee et al., segmental meniscectomy (i.e., loss of hoop tension) was shown to be equivalent to total meniscectomy in load-bearing terms. The authors also observed that the peripheral portion of the medial meniscus played a more significant role in decreasing mean contact stresses and increasing contact areas than the central portion, whereas the amount of meniscus removed proportionally increased peak contact stresses [32].

In 2016, Koh et al., using Tekscan sensors, studied tibiofemoral contact pressure and contact area of the medial meniscus with horizontal cleavage tear (HCT) and resection. Performing five serial conditions of posterior medial meniscectomy (intact, HCT, repaired HCT, inferior leaf resection, and resection of both inferior and superior leaves) at 0- and 60-degree knee flexion angles under an 800-N axial load demonstrated that resection of the inferior leaf resulted in significantly decreased contact area and increased peak contact pressure in the medial compartment [33]. Thereupon, a more comprehensive study for assessing the changes in the tibiofemoral contact pressure and contact area was performed in a HCT before and after surgery by Beamer et al. [34]. Knees were mounted on a customized jig and loaded twice the body weight while using pressure sensors under the medial meniscus. Contact pressure and contact area were recorded at 0°, 10°, and 20° of flexion, performing 20 cycles of axial loading at the rate of 1 Hz. HCT in the medial meniscus produced a significant decrease in the contact area and a significant rise in contact pressure, which may stimulate joint degeneration. In contradiction, both partial and subtotal meniscectomy caused significant reduction in the contact area and significant elevations in contact pressure in the knee.

### FE modeling studies

**Fig. 5:**
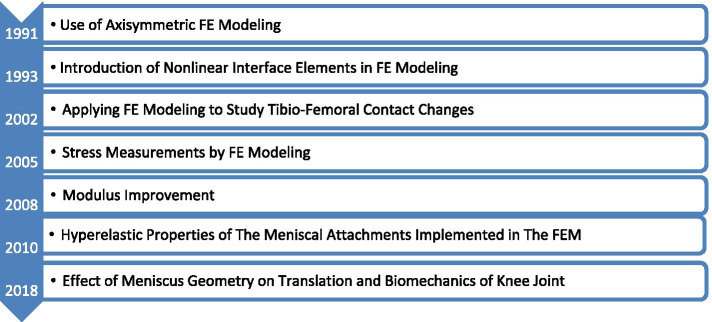
Milestones in finite element modeling of the meniscus

Numerical simulations have recently gained significance in biomechanical research. Multibody dynamics is a numerical approach that deals with the kinematics of musculoskeletal activities. Another major numerical approach is structure–mechanical point-of-view, which deals with the localized stress–strain analysis of bones, joints (both natural and artificial), and bodies that exhibit load-bearing capacity. FE models employing these numerical and simulative approaches started to gain momentum in the 1990s with biomechanical analyses of the knee and the meniscus [8–10]. Generating reliable, accurate, and meaningful FE models required investigators to understand the material properties of all articular tissues of the knee such that the results could be compared to cadaveric findings, the gold standard at the time [10–12]. Figure 5 outlines the FE studies involving the meniscus since its early use in the 1990s.

One such study that first demonstrated the utility of FE modeling for understanding the meniscus’ biomechanical properties involved an axisymmetric FE model comprised of a rigid spherical indentor, meniscal ring, and an articular cartilaginous layer interacting with an ideal fluid sub-system to evaluate the transmission of force during tibiofemoral contact [9]. The authors observed that the fluid in the cavity endured the largest proportion of the simulated load initially, while toward the end of the simulation, the meniscal ring bore the major part of the load. It was also shown that the indentor’s curvature appeared to mediate a notable effect on the load-bearing of the meniscal ring immediately after stepwise changes of loading were applied and immediately disappeared as fluid started to exude from the model [10]. Soon after, Lengsfeld investigated stress distributions at the meniscofemoral joint in 1993 by analyzing the applicability of nonlinear interface elements in a FE model. The analysis revealed that two force transfer peaks—intercondylar border and femoral center—were mediated by a decrease in the elastic modulus of the articular surface tissues. The modeled width of the gap between femoral cartilage and meniscus or tibial cartilage had a strong influence on those two force transfer peaks [8].

Advances in computational power have mediated more resolved mesh density for FE models over the past 20 years, as FE modeling has become a popular approach for characterizing biomechanical aspects of the meniscus. Donahue et al. developed a FE model suitable to study tibiofemoral contact changes after meniscal replacement, which included cortical and trabecular bone of the femur and tibia, cartilaginous articular tissue of the tibial plateau and femoral condyles, medial and lateral menisci with their corresponding horn attachments, transverse ligaments, anterior cruciate ligament, and medial collateral ligament. This group considered meniscal tissue as transversely isotropic and linearly elastic, making analyses under the presence of an 800 N compressive load at 0**°** of flexion [35].

In a study by Meakin et al., FE analysis was used to demonstrate that femoral-meniscal curvature mismatches could have a large effect on the stresses on both surfaces and thus should correspondingly be considered when developing meniscal repair or replacement interventions [36]. Biomechanical pathways lead to two types of damage to the cartilage; type 1- damage without disruption of the underlying bone or calcified cartilage layer or type 2- subchondral fracture with or without damage to the overlying cartilage. These damages cause degeneration after meniscectomy, described by Wilson et al. in 2003 using FE modeling [37]. Later in 2005, FE analysis was utilized to measure maximum contact stress for the posterior zone of the medial meniscus after meniscectomy in zero degree flexion, showing a doubling of stress post-procedure [38]. The following year, the same group used FE to demonstrate that under axial femoral compressive loads, the maximum shear stress and peak contact stress in the articular cartilage increased twice as much following a lateral meniscectomy compared with a medial meniscectomy [39]. Yao et al. identified the initial strain of the meniscal horn attachments (epsilon (1H) = –5%), the ratio of meniscal moduli in the circumferential and transverse directions (E (theta) E(R) = 20), and the linear modulus of the meniscal peripheral attachments (E (P) = 5.6 MPa), in order to minimize FE model errors. The influence of inhomogeneity and nonlinear properties in developing accurate and working FE models was also reported in this study [40].

**Fig. 6:**
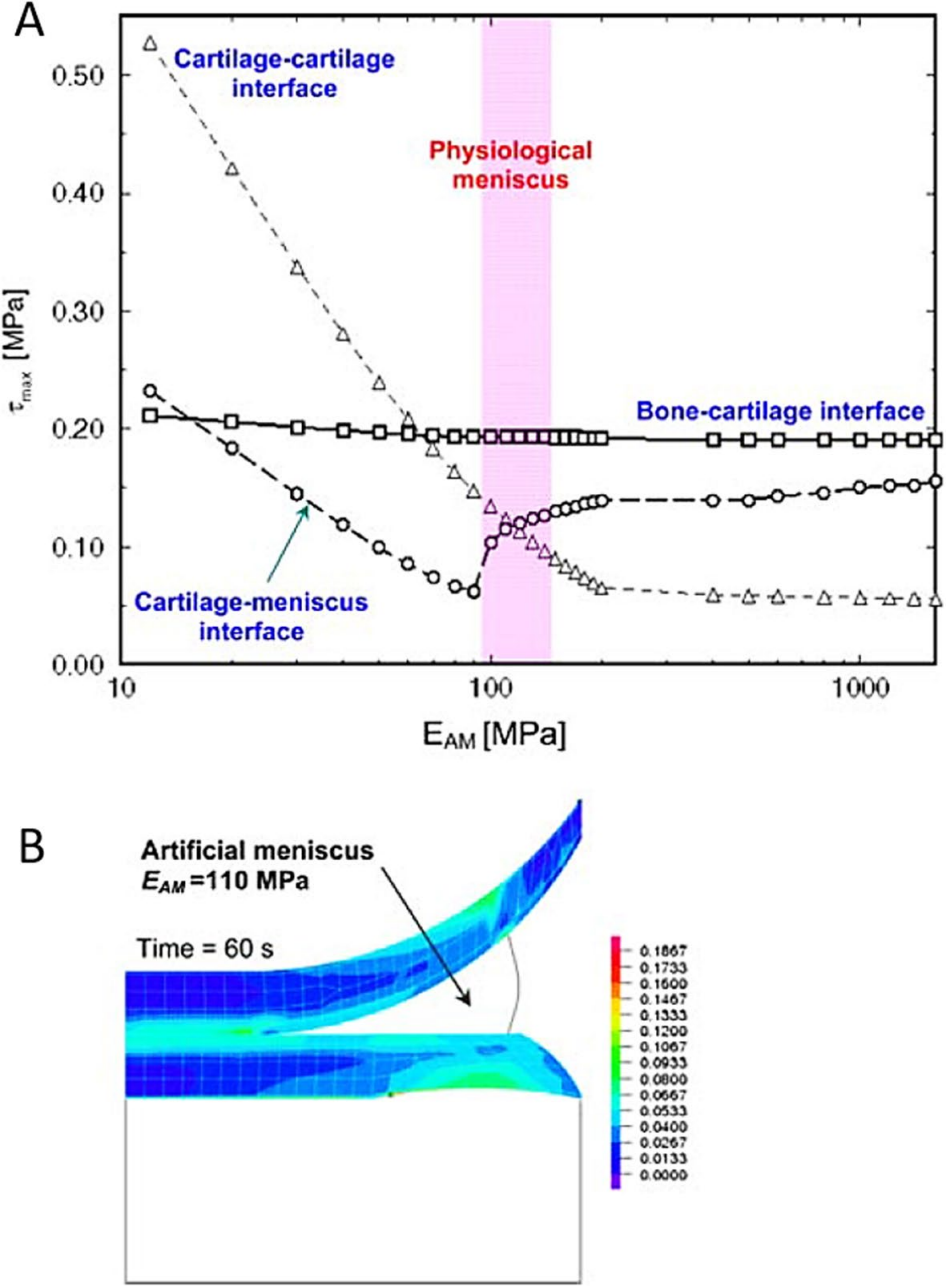
The distribution of shear stresses in the articular cartilage. **A** Influence of the Young’s modulus of the artificial meniscus, dented by EAM, on the maximum shear stresses at the interfaces of cartilage–cartilage, cartilage–bone, and cartilage–meniscus. A range for the stiffness of physiological meniscus in the circumferential direction of the knee is also shown. **B** Shear stress distribution in the auricular cartilages with artificial meniscus with EAM = 110 MPa. The Poisson ratio of the artificial meniscus is equal to 0.45 in this set of calculations. From “Influence of meniscectomy and meniscus replacement on the stress distribution in human knee joint.”, by Vaziri A et al., 2008, Ann Biomed Eng. 2008;36(8):1335–44 [45]. With permission of Springer

**Fig. 7:**
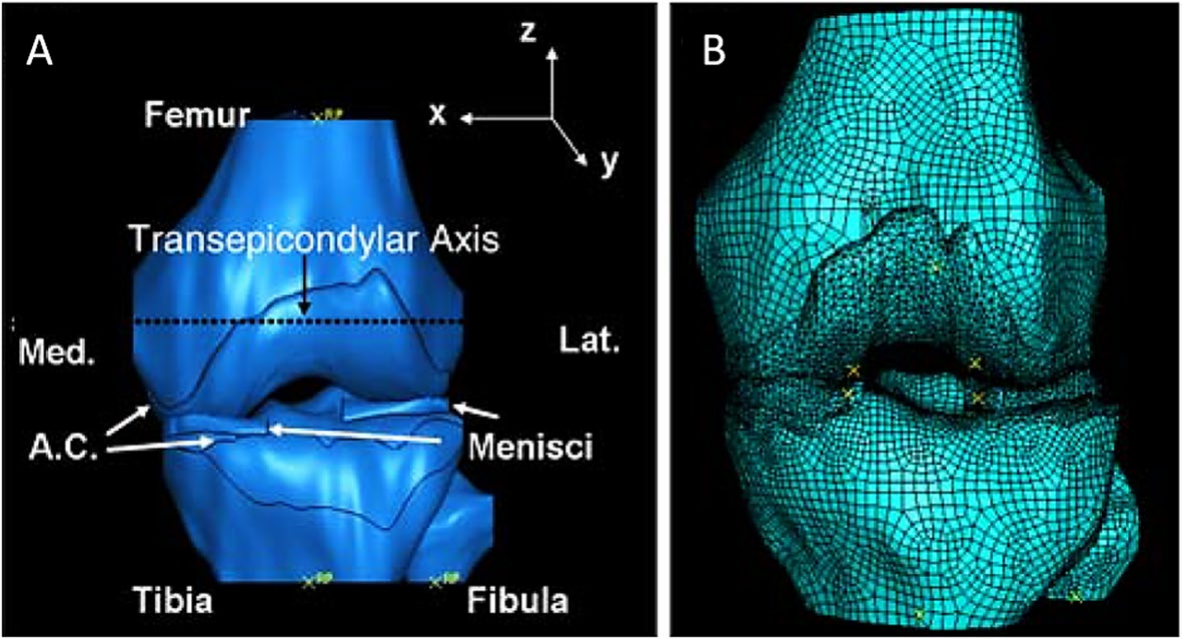
**A** Three-dimensional geometry of the left knee, which includes femur, tibia, fibula, articular cartilage and lateral and medial menisci with the dotted line representing the trans epicondylar axis and the location where the loading was applied. **B** A typical mesh of the knee geometry. From “The combined effect of frontal plane tibiofemoral knee angle and meniscectomy on the cartilage contact stresses and strains.”, by Yang N et al., 2009, Ann Biomed Eng. 2009;37(11):2360–72 [46]. With permission of Springer

Vadher et al. conducted further understanding of the effect of resection size in partial meniscectomy provided by cadaveric studies in 2006 that utilized FE modeling [41]. They showed that removing greater than 20% of the meniscus, drastically increased the stresses experienced in the knee, and a 65% meniscectomy resulted in a 225% increase in maximal shear stress in the cartilage when compared to normal. These studies continued by using a 3D FE model to evaluate the effects of various locations and extent of meniscectomy on tibial articular [42, 43]. Atmaca et al. confirmed the previous studies by showing that the extent of meniscectomy plays an essential role in tibial articular cartilage load. They also showed that longitudinal meniscectomies of more than 25% lead to more stress than anterior or posterior meniscectomy [44]. Among several tested conditions, Dong et al. confirmed the previous findings that longitudinal meniscectomy and oblique tears led to the largest values of the peak compressive and shear stresses on tibial articular cartilage [43]. In 2008, Vaziri et al., using FE modeling, suggested 110 MPa as an optimal elastic modulus for the artificial meniscus (Fig. 6) [45]. The determination of optimal elastic modulus would allow more complex and clinically relevant investigations of artificial menisci for implant design and to evolve querying biomechanical outcomes of the knee. Further FE model investigations by Yang et al. in 2009 and 2010 demonstrated that an individual’s frontal plane knee alignment was an essential factor when considering the effect of total or partial meniscectomy on the biomechanics of the knee (Fig. 7) [46, 47].

In 2010, Abraham et al. studied the hyperelastic properties of meniscal attachments to revise and improve the overall design of FE model studies. In Particular, this group set out to evaluate the tensile mechanical properties of meniscal attachments in the transverse direction and curve fit experimental Cauchy stress–strain data to consider hyperelastic behavior. Moreover, these authors coupled those results with previously obtained longitudinal data to create a more complete constitutive model of meniscal behavior. They demonstrated that medial posterior attachments, when compared to the other attachments via curve fitting correlation, had a significantly higher elastic modulus (6.42 ± 0.78 MPa) and ultimate stress (1.73 ± 0.32 MPa) [48]. Studies on the composition of the meniscus and its effect on load absorption continued in 2014 when Parraga Quiroga et al. used an FE model to evaluate whether the meniscus’ depth-dependent matrix composition is essential for its mechanical behavior. They showed that knee joint mechanics are not as sensitive to the distribution of constitutive components in the cross-section of the meniscus. Thus, depth-dependent matrix distribution is dispensable in the axisymmetric computational models of the knee joint [49]. Using the same modeling approach, this team studied the effect of geometrical mismatches on menisci’s chondroprotective capabilities [50]. They showed that strains inside the articular cartilage strongly depended on loading duration and implant size rather than their stiffness or material.

In 2018, Luczkiewicz et al. used an MRI-based FE of the knee to model varying meniscus heights and cross-sectional shapes under a compressive load of 1000 N in order to assess the influence of meniscus geometry on translation and biomechanics of the knee joint. They found that the meniscus external shift was affected by the meniscus geometry and changes in cross-section. This finding is significant, considering that meniscal extrusion may decrease mechanical protection of the surrounding cartilage. Moreover, they concluded that changes in meniscus geometry affect the knee joint’s congruity and its medio-lateral translation [51].

### Kinematic studies

**Fig. 8:**
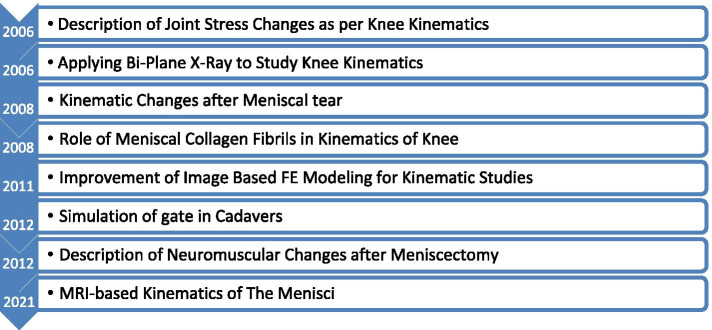
Significant events in kinematic studies of the meniscus

The new millennium enhanced cadaveric and FE modeling by combining their separate but complementary utilities toward in-vivo conditions*.* Kinematic studies addressed numerous biomechanical and clinically relevant questions regarding stress (mechanical) changes in the meniscus during various dynamic movements of the knee (Fig. 8).

The effect of knee kinematics in tibio-menisco-femoral joint contact properties was investigated by Yao et al. using FE analysis in 2006, which illuminated the need to improve the precision and accuracy of knee kinematic measurements in order to make FE modeling analysis a reliable tool [40]. This improvement was heeded by Papaioannou et al., who used biplane dynamic Roentgen stereogrammetric analysis to validate their patient-specific knee joint FE model in 2008 to enhance model fidelity at the level of contact between menisci and cartilage tissue [52]. This study addressed the problem of accuracy and precision of kinematic measurements, which was indicated earlier in 2006 [40]. Papaioannou et al. conducted another test where they changed the mesh size to 1 × 1 mm elements, increasing the magnitude of contact variables by up to 45% [52].

The knee joint’s biomechanical properties with tears in the posterior root of the medial meniscus and subsequent repair mechanisms were studied by Allair et al. and Harner et al. [53, 54]. The clinical significance of these tears was known to cause rapid and progressive arthritis; however, their biomechanical effects were not apparent until the latter years of the last decade. These studies showed that contact pressures increased in both lateral and medial sides due to posterior root tear of the medial meniscus [54]. An investigation into the role of the cartilage collagen fibril network in the biomechanics of the knee was conducted by Shirazi et al. in 2008, who reported that under transient compression, deep fibrils, and to a lesser extent superficial fibrils, played major mechanical roles in cartilage response [55].

In 2011, Haemer et al. introduced a new image-based FE model using imaging data and a contact indenter boundary condition approach based on changes in articular cartilage mechanics, concurrent with observed deformation [56]. In 2012, Seitz et al. investigated the loads acting on the anterior menisco-tibial attachments under eight conditions with subsequent axial loading at 0°, 30°, and 60° of knee flexion [57]. This study suggested that contact mechanics were likely to be more sensitive to partial meniscectomy at higher flexion angles [57]. Using the same setting, the authors addressed the effect of partial meniscectomy in various degrees of flexion in the knee. They showed that when the knee is flexed up to 30°, both 20 and 50% partial meniscectomy would not increase maximum contact pressure or decrease the contact area. However, at 60° of flexion, a 50% partial meniscectomy would increase contact pressure. Furthermore, total meniscectomy would affect the mechanics of the joint significantly regardless of the flexion angle [57].

**Fig. 9:**
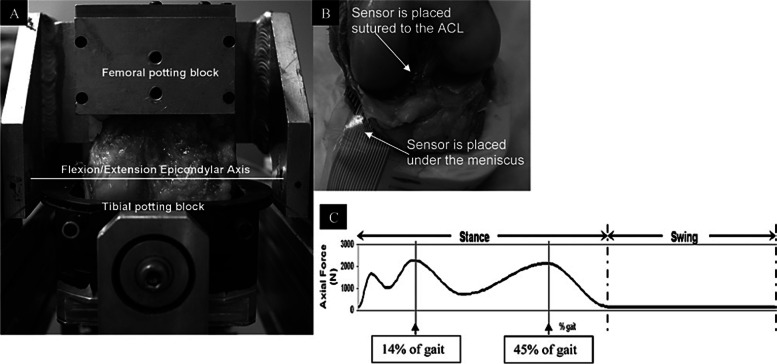
Kinematic study of radial tears of the lateral meniscus and its repair. The contact mechanics of large radial lateral meniscus tears were similar to those of partial lateral meniscectomy but contact pressure was significantly reduced with inside-out repair. **A** Stanmore Knee Simulator, **B** Sensor Placement, **C** The Force Profile. From “Dynamic contact mechanics of radial tears of the lateral meniscus: implications for treatment.”, by Bedi A et al., 2012, Arthroscopy. 2012;28(3):372–81 [58]. Copyright (2012), with permission from Elsevier

In 2012, Bedi et al. employed simulated human gait in cadaveric specimens to study the biomechanical effects of radial lateral meniscus tears (Fig. 9) [58]. Conversely, increased contact pressure and reduced contact area in the patellofemoral joint were observed by Bai et al. in a cadaveric study after performing increasing degrees of meniscectomies [59]. Thorlund et al. studied the neuromuscular function of meniscectomized patients and found a reduced range of motion, increased muscle coactivation, and increased loading rate in the operated leg compared with the contralateral leg in that group. They concluded that those findings might precede and affect the development of osteoarthritis [60]. In a recent study using an open-structure MRI, meniscal kinematics were investigated in a full knee range of motion. This study demonstrated that meniscal kinematics were analogs to the femorotibial kinematics. This comprehension could help us understand the mechanism of injury and plan for the surgery or post-surgical rehabilitation programs [61].

## Conclusion

Early studies focused primarily on cadaveric models, which allowed investigators to empirically determine the effects of meniscal tissue statically in normal or pathologic conditions, and also pseudo-dynamically through load-bearing simulations. By late 1986, the biomechanical characteristics of the meniscus after degrees of meniscectomy had been extensively studied. These studies showed the importance of cartilaginous meniscal tissue for protecting the articular tibial surface during axial compressive forces as well as for tibiofemoral stabilization. The importance of meniscal preservation followed this comprehension; first through the perspective of cadaveric models and then followed by FE models.

The biomechanics of intact meniscus and various types of meniscal tears in the healthy knee has been broadly studied; however, some challenges and controversies have yet to be addressed. The development or improvement of surgical techniques guarantees the continuation of such studies. Nevertheless, the biomechanics of meniscal tears, meniscectomy, or repairs in the knee with other concurrent problems such as torn cruciate ligaments or genu-valgum or genu-varum have not been extensively studied. Furthermore, using FE modeling to understand the effect of meniscal tears in athletes or workers in extreme conditions would demonstrate whether there is an indication for meniscal repair or meniscectomy in such circumstances.

## Data Availability

The datasets used and/or analysed during the current study are available from the corresponding author on reasonable request.

## Abbreviations

MeSH: Medical Subject Headings
FE: Finite Element
ACL: Anterior cruciate ligament

## References

[1] Ahmed AM, Burke DL (1983). In-vitro measurement of static pressure distribution in synovial joints–part I: tibial surface of the knee. J Biomech Eng.

[2] Fithian DC, Kelly MA, Mow VC (1990). Material properties and structure-function relationships in the menisci. Clin Orthop Relat Res.

[3] Vedi V, Williams A, Tennant SJ, Spouse E, Hunt DM, Gedroyc WM (1999). Meniscal movement. An in-vivo study using dynamic MRI. J Bone Joint Surg Br.

[4] Amendola A, Bonasia DE (2012). The menisci: anatomy, healing response, and biomechanics. The knee joint: surgical techniques and strategies.

[5] Bland Sutton J. Ligaments: their nature and morphology. Bristol Med Chir J (1883). 1897;15(58):344.

[6] Rodeo SA, Monibi F, Dehghani B, Maher S (2020). Biological and mechanical predictors of meniscus function: basic science to clinical translation. J Orthop Res.

[7] Frankel VH, Burstein AH, Brooks DB (1971). Biomechanics of internal derangement of the knee. Pathomechanics as determined by analysis of the instant centers of motion. J Bone Joint Surg Am.

[8] Lengsfeld M (1993). Stresses at the meniscofemoral joint: elastostatic investigations on the applicability of interface elements. J Biomed Eng.

[9] Schreppers GJ, Sauren AA, Huson A (1990). A numerical model of the load transmission in the tibio-femoral contact area. Proc Inst Mech Eng H.

[10] Schreppers GJ, Sauren AA, Huson A (1991). A model of force transmission in the tibio-femoral contact incorporating fluid and mixtures. Proc Inst Mech Eng H.

[11] Goertzen DJ, Budney DR, Cinats JG (1997). Methodology and apparatus to determine material properties of the knee joint meniscus. Med Eng Phys.

[12] Tissakht M, Ahmed AM (1995). Tensile stress-strain characteristics of the human meniscal material. J Biomech.

[13] Kennedy JC, Fowler PJ (1971). Medial and anterior instability of the knee. An anatomical and clinical study using stress machines. J Bone Joint Surg Am.

[14] Hsieh HH, Walker PS (1976). Stabilizing mechanisms of the loaded and unloaded knee joint. J Bone Joint Surg Am.

[15] Bylski-Austrow DI, Ciarelli MJ, Kayner DC, Matthews LS, Goldstein SA (1994). Displacements of the menisci under joint load: an in vitro study in human knees. J Biomech.

[16] Ikeuchi K, Sakoda H, Sakaue R, Tsuji K, Tomita N (1998). A new method for accurate measurement of displacement of the knee menisci. Proc Inst Mech Eng H.

[17] Tienen TG, Buma P, Scholten JG, van Kampen A, Veth RP, Verdonschot N (2005). Displacement of the medial meniscus within the passive motion characteristics of the human knee joint: an RSA study in human cadaver knees. Knee Surg Sports Traumatol Arthrosc.

[18] Uezaki N, Kobayashi A, Matsushige K (1979). The viscoelastic properties of the human semilunar cartilage. J Biomech.

[19] Egner E (1982). Knee joint meniscal degeneration as it relates to tissue fiber structure and mechanical resistance. Pathol Res Pract.

[20] Petersen W, Tillmann B (1998). Collagenous fibril texture of the human knee joint menisci. Anat Embryol (Berl).

[21] Bursac P, York A, Kuznia P, Brown LM, Arnoczky SP (2009). Influence of donor age on the biomechanical and biochemical properties of human meniscal allografts. Am J Sports Med.

[22] Gaugler M, Wirz D, Ronken S, Hafner M, Gopfert B, Friederich NF (2015). Fibrous cartilage of human menisci is less shock-absorbing and energy-dissipating than hyaline cartilage. Knee Surg Sports Traumatol Arthrosc.

[23] Walker PS, Erkman MJ (1975). The role of the menisci in force transmission across the knee. Clin Orthop Relat Res.

[24] Radin EL, de Lamotte F, Maquet P (1984). Role of the menisci in the distribution of stress in the knee. Clin Orthop Relat Res.

[25] Bourne RB, Finlay JB, Papadopoulos P, Andreae P (1984). The effect of medial meniscectomy on strain distribution in the proximal part of the tibia. J Bone Joint Surg Am.

[26] Brown TD, Shaw DT (1984). In vitro contact stress distribution on the femoral condyles. J Orthop Res.

[27] Baratz ME, Fu FH, Mengato R (1986). Meniscal tears: the effect of meniscectomy and of repair on intraarticular contact areas and stress in the human knee. A preliminary report. Am J Sports Med.

[28] Ihn JC, Ahn MW, Kim DM (1992). Photoelastic analysis of stress distribution on the tibiofemoral joint after meniscectomy. Orthopedics.

[29] Ihn JC, Kim SJ, Park IH (1993). In vitro study of contact area and pressure distribution in the human knee after partial and total meniscectomy. Int Orthop.

[30] Lechner K, Hull ML, Howell SM (2000). Is the circumferential tensile modulus within a human medial meniscus affected by the test sample location and cross-sectional area?. J Orthop Res.

[31] Leslie BW, Gardner DL, McGeough JA, Moran RS (2000). Anisotropic response of the human knee joint meniscus to unconfined compression. Proc Inst Mech Eng H.

[32] Donell ST, Marshall TJ, Darrah C, Shepstone L (2006). Cruciate ligament assessment in MRI scans: a pilot study of a static drawer technique. Knee.

[33] Koh JL, Yi SJ, Ren Y, Zimmerman TA, Zhang LQ (2016). Tibiofemoral contact mechanics with horizontal cleavage tear and resection of the medial meniscus in the human knee. J Bone Joint Surg Am.

[34] Beamer BS, Walley KC, Okajima S, Manoukian OS, Perez-Viloria M, DeAngelis JP (2017). Changes in contact area in meniscus horizontal cleavage tears subjected to repair and resection. Arthroscopy.

[35] Donahue TL, Hull ML, Rashid MM, Jacobs CR (2002). A finite element model of the human knee joint for the study of tibio-femoral contact. J Biomech Eng.

[36] Meakin JR, Shrive NG, Frank CB, Hart DA (2003). Finite element analysis of the meniscus: the influence of geometry and material properties on its behaviour. Knee.

[37] Wilson W, van Rietbergen B, van Donkelaar CC, Huiskes R (2003). Pathways of load-induced cartilage damage causing cartilage degeneration in the knee after meniscectomy. J Biomech.

[38] Pena E, Calvo B, Martinez MA, Palanca D, Doblare M (2005). Finite element analysis of the effect of meniscal tears and meniscectomies on human knee biomechanics. Clin Biomech (Bristol, Avon).

[39] Pena E, Calvo B, Martinez MA, Palanca D, Doblare M (2006). Why lateral meniscectomy is more dangerous than medial meniscectomy. A finite element study. J Orthop Res.

[40] Yao J, Funkenbusch PD, Snibbe J, Maloney M, Lerner AL (2006). Sensitivities of medial meniscal motion and deformation to material properties of articular cartilage, meniscus and meniscal attachments using design of experiments methods. J Biomech Eng.

[41] Vadher SP, Nayeb-Hashemi H, Canavan PK, Warner GM (2006). Finite element modeling following partial meniscectomy: effect of various size of resection. Conf Proc IEEE Eng Med Biol Soc.

[42] Atmaca H, Ozkan A, Mutlu I, Celik T, Ugur L, Kisioglu Y (2014). The effect of proximal tibial corrective osteotomy on menisci, tibia and tarsal bones: a finite element model study of tibia vara. Int J Med Robot.

[43] Dong Y, Hu G, Dong Y, Hu Y, Xu Q (2014). The effect of meniscal tears and resultant partial meniscectomies on the knee contact stresses: a finite element analysis. Comput Methods Biomech Biomed Engin.

[44] Atmaca H, Kesemenli CC, Memisoglu K, Ozkan A, Celik Y (2013). Changes in the loading of tibial articular cartilage following medial meniscectomy: a finite element analysis study. Knee Surg Sports Traumatol Arthrosc.

[45] Vaziri A, Nayeb-Hashemi H, Singh A, Tafti BA (2008). Influence of meniscectomy and meniscus replacement on the stress distribution in human knee joint. Ann Biomed Eng.

[46] Yang N, Nayeb-Hashemi H, Canavan PK (2009). The combined effect of frontal plane tibiofemoral knee angle and meniscectomy on the cartilage contact stresses and strains. Ann Biomed Eng.

[47] Yang N, Canavan P, Nayeb-Hashemi H, Najafi B, Vaziri A (2010). Protocol for constructing subject-specific biomechanical models of knee joint. Comput Methods Biomech Biomedi Eng.

[48] Abraham AC, Moyer JT, Villegas DF, Odegard GM, Haut Donahue TL (2011). Hyperelastic properties of human meniscal attachments. J Biomech.

[49] ParragaQuiroga JM, Emans P, Wilson W, Ito K, van Donkelaar CC (2014). Should a native depth-dependent distribution of human meniscus constitutive components be considered in FEA-models of the knee joint?. J Mech Behav Biomed Mater.

[50] ParragaQuiroga JM, Ito K, van Donkelaar CC (2015). Meniscus replacement: Influence of geometrical mismatches on chondroprotective capabilities. J Biomech.

[51] Luczkiewicz P, Daszkiewicz K, Witkowski W, Chroscielewski J, Ferenc T, Baczkowski B (2018). The influence of a change in the meniscus cross-sectional shape on the medio-lateral translation of the knee joint and meniscal extrusion. PLoS One.

[52] Papaioannou G, Nianios G, Mitrogiannis C, Fyhrie D, Tashman S, Yang KH (2008). Patient-specific knee joint finite element model validation with high-accuracy kinematics from biplane dynamic Roentgen stereogrammetric analysis. J Biomech.

[53] Allaire R, Muriuki M, Gilbertson L, Harner CD (2008). Biomechanical consequences of a tear of the posterior root of the medial meniscus. Similar to total meniscectomy. J Bone Joint Surg Am.

[54] Harner CD, Mauro CS, Lesniak BP, Romanowski JR (2009). Biomechanical consequences of a tear of the posterior root of the medial meniscus. Surgical technique. J Bone Joint Surg Am.

[55] Shirazi R, Shirazi-Adl A, Hurtig M (2008). Role of cartilage collagen fibrils networks in knee joint biomechanics under compression. J Biomech.

[56] Haemer JM, Song Y, Carter DR, Giori NJ (2011). Changes in articular cartilage mechanics with meniscectomy: a novel image-based modeling approach and comparison to patterns of OA. J Biomech.

[57] Seitz AM, Lubomierski A, Friemert B, Ignatius A, Durselen L (2012). Effect of partial meniscectomy at the medial posterior horn on tibiofemoral contact mechanics and meniscal hoop strains in human knees. J Orthop Res.

[58] Bedi A, Kelly N, Baad M, Fox AJ, Ma Y, Warren RF (2012). Dynamic contact mechanics of radial tears of the lateral meniscus: implications for treatment. Arthroscopy.

[59] Bai B, Shun H, Yin ZX, Liao ZW, Chen N (2012). Changes of contact pressure and area in patellofemoral joint after different meniscectomies. Int Orthop.

[60] Thorlund JB, Damgaard J, Roos EM, Aagaard P (2012). Neuromuscular function during a forward lunge in meniscectomized patients. Med Sci Sports Exerc.

[61] Yamamoto T, Taneichi H, Seo Y, Yoshikawa K. MRI-based kinematics of the menisci through full knee range of motion. J Orthop Surg (Hong Kong). 2021;29(2):23094990211017348. 10.1177/23094990211017349 34027726

